# eImmunonkologie: Development and Launch of a Virtual Education Platform for the Immunotherapy of Cutaneous Neoplasms

**DOI:** 10.1007/s40670-022-01713-8

**Published:** 2022-12-20

**Authors:** Matthias D. Kaufmann, Theresa Steeb, Anja Wessely, Marion Meyerolbersleben, Lars E. French, Carola Berking, Markus V. Heppt

**Affiliations:** 1 Department of Dermatology, Uniklinikum Erlangen, Friedrich-Alexander-Universität Erlangen-Nürnberg (FAU), Ulmenweg 18, 91054 Erlangen, Germany; 2 Comprehensive Cancer Center Erlangen - Europäische Metropolregion Nürnberg (CCC ER-EMN), Erlangen, Germany; 3 Institut für Lern-Innovation (ILI), Friedrich-Alexander-Universität Erlangen-Nürnberg (FAU), Fürth, Germany; 4 Department of Dermatology, Klinikum der Universität München, Ludwig-Maximilian University München, Munich, Germany

**Keywords:** Immunotherapy, Skin cancer, Medical education, eLearning, Immuno-oncology, Virtual education platform

## Abstract

The use of immunotherapies in clinical practice has significantly expanded treatment options and improved the prognosis of patients with advanced cancers over the past decade. We have developed a virtual teaching module entitled “eImmunonkologie” which is the first interdisciplinary virtual course on immuno-oncology for medical students in German-speaking countries.

The use of immunotherapy in clinical practice has greatly enriched the oncological treatment options and improved the prognosis of patients with advanced skin cancers over the past decade. Given the increasing spread and use of immunotherapies also in other entities, it is very important to introduce the next generation of physicians to this topic at an early stage of education. Unfortunately, immunotherapy has not been adequately represented and situated in medical education in Germany yet. For example, the topics of immuno-oncology or checkpoint blockade have never been addressed in written medical examinations [1]. Physicians of all specialties should have a basic understanding of immunotherapies, their opportunities but also their risks, and be able to recognize potentially fatal side effects in order to appropriately treat and counsel patients. Therefore, we developed and launched a virtual teaching module entitled “eImmunonkologie” for medical students as well as physicians in training.

The course aims to provide interdisciplinary basic knowledge on immunotherapy with a focus on cutaneous neoplasms. The content includes seven sequential chapters (Fig. 1A) and starts after a section with general course information dealing with the history of immunotherapy from its conception to its current clinical use, the basic mechanisms of action and tumor immunology, how checkpoint inhibitors work, and the disease entities for which the therapy is currently approved (Fig. 1B). At the same time, the course also educates on common adverse events and their management which is highly important for physicians working primarily in oncology, but also in outpatient care. The content was created by two physicians who prepared the topic in an understandable way using the most recent literature and research results. For students who are interested in more details, corresponding papers are attached to each chapter. The teaching module is complemented by several interactive case studies to consolidate and directly apply the acquired knowledge. For this purpose, real patient cases from our clinical practice are used, e.g., a case of severe upper abdominal pain and suspected liver metastases from melanoma with subsequent treatment or a case of an emergency presentation with somnolence and fatigue under already ongoing immunotherapy. The course participants must then independently determine the further diagnostic and therapeutic path in each case. Finally, they receive feedback on the decisions made (Fig. 1C).

**Fig. 1:**
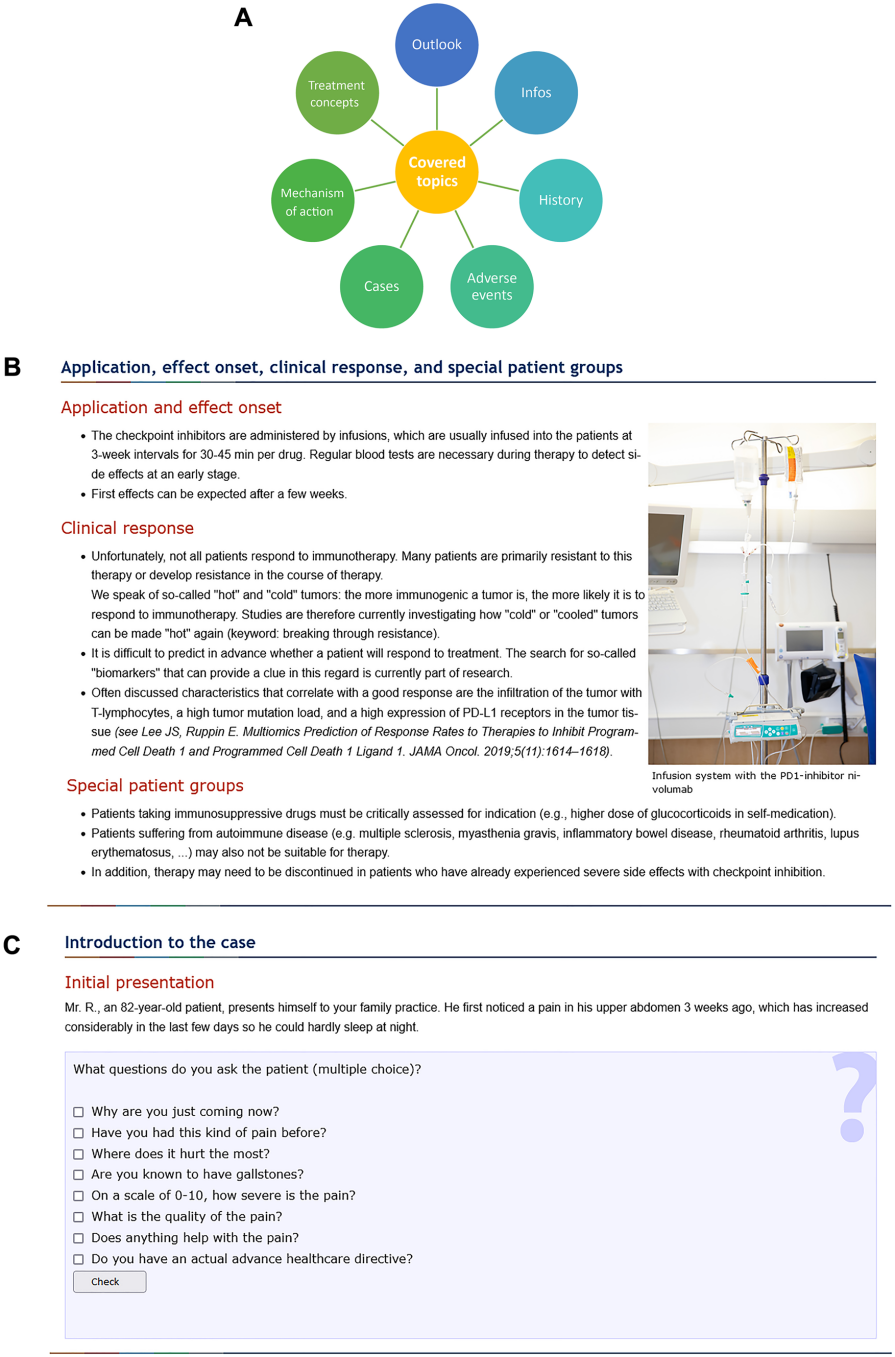
(**A**) Module overview of the “eImmunonkologie” virtual education platform. (**B**) Example slide of the module “Checkpoint inhibitors” (translated). (**C**) Illustrative learning success checks of the interactive case studies (translated)

Successful completion of the course requires finishing several exams (e.g., single or multiple choice questions, gap texts) after each chapter and a digital essay of a patient case which must be submitted separately followed by individual correction and feedback regarding learning potential. This blended review approach controls the newly acquired knowledge and exploits the potential of digital teaching through interactive question formats. The course is integrated as a thematic and voluntary supplement into the main lecture “Dermatology” at the Friedrich-Alexander-Universität Erlangen-Nürnberg (FAU) in the 4th year of medical school. Participation independent of this lecture is also possible. Upon successful completion, participants receive two bonus points for the written examination; partial half points are also awarded.

The module was technically implemented on the digital learning management system entitled “ILIAS” with the integration of interactive plug-ins (e.g., an examination plug-in called “H5P”). We also enabled the integration of social media functions such as a forum for the interaction of participants moderated by a physician of our team. The development of the course was financially supported by the “Virtuelle Hochschule Bayern e.V.” (VHB) which hosts the course. We were advised on media didactics by the “Institut für Lern-Innovation (ILI)” at the FAU with profound knowledge about the creation of virtual teaching formats [2].

The course was launched in the winter term 2021 and has been successfully completed by nearly 350 medical students so far. The evaluation shows a high level of interest in this topic and we receive consistently positive feedback. Many students confirmed our thesis that immunotherapy is currently only rarely addressed in medical school.

To the best of our knowledge, the development of this course is the first interdisciplinary and freely accessible virtual course on immuno-oncology for medical students, as well as physicians in training in German-speaking countries. However, it can be completed by any interested parties from other universities after prior registration to the VHB. Continuous evaluations and further development of the course content (e.g., multilingual content) are planned.

Have we aroused your interest? Access to the course is possible via the website of the “Virtuelle Hochschule Bayern e.V.” (www.vhb.org > Classic vhb > Dermatologie > eImmunonkologie).

## Data Availability

Not applicable.
